# Relapse of incisor crowding: A visit to the prince of salina

**DOI:** 10.4317/medoral.18514

**Published:** 2012-12-10

**Authors:** Luis López-Areal, Jose L. Gandía

**Affiliations:** 1 Orthodontist. Master in Orthodontics. University of Valencia. Associate Professor. Faculty of Medicine and Dentistry, Universidad del País Vasco (UPV/EHU); 2 Orthodontist. Full Professor of Dentistry (Orthodontics Teaching Unit). Director of Master in Orthodontics, University of Valencia; 3 ….

## Abstract

The management of the retention period after comprehensive orthodontic treatment is of great importance, as a primary goal of clinician. Considerable controversy still surrounds the problem of stability after the retention period. Many studies analyze factors associated to the presence of crowding or incisor irregularity and find predictive features on its relapse. Most studies have reported little o no correlation between the treatment changes in the biological parameters - clinical, biometric (irregularity index, intermolar width, intercanine width, arch length, overjet, overbite), or cephalometric variables- that ocurred and the posttretament and postretention changes that may predict their future development. This article provides a bibliographical overview on the relapse of dental alignment in treated cases. In a brief historical introduction, the first studies on the long-term stability of orthodontic results are analysed. The article then goes on to assess studies that focus attention on anteroinferior alignment before finally studying relapse of upper crowding. It concludes by making some final comments in the light of the bibliography provided and the differents schools regarding retention needs and methods.

** Key words:**Retention, stability, irregularity, dental alignment.

## Introduction

When we check up on treated cases some years after the end of maintenance and compare initial records with post-retention records, we are reminded of the well-known saying everything must change if we want things to remain as they are, which appears in “The Leopard”, an excellent novel by Lampedusa and adapted for cinema by Visconti in a magnificent film of the same title. With this statement, the Prince of Salina, an aging Sicilian aristocrat, is reprimanded by his nephew as they face the new era that was to usher in Italian unification.

This sense of immutability also emanates from our store of boxes of plaster casts together with their dust and yellowing labels.

Indeed, we can then check that on no few occasions the initial malocclusion returns once again to the plaster after a brief period of orthodontic correction. The relapse/stability binomial is a fragile balance that we are often unable to tilt in our favour. Why does this happen? From Angle’s time relapse has been a real headache for the profession, especially as far as anterior alignment is concerned, due to the aesthetic and social importance that having a nice smile carries with it.

The aim of this work is to review part of what has been written on the relapse of incisor crowding since then to the present date.

## Historical Antecedents

One of the aims of all orthodontic treatments, apart from achieving an acceptable aesthetic and functional result, is the long-term stability.

Orthodontic correction implies the unleashing of several powerful mechanical stimuli on the periodontal tissues, changes to which allow tooth movement. On eliminating these stimuli, the tissues have to readapt to this new tooth position and this accommodation generates several tensions that tend to mobilize the tooth once again, partially thwarting the work of the orthodontist. It is for this reason that it is essential to fix this new position for a certain time, by implementing what is known as the retention stage.

Relapse is a concept that is antagonistic to stability and can be defined in Orthodontics as the tendency of treated teeth to return to their original position, as it may be assumed that in most malocclusions, the final positions that the pieces have reached over the years are those that provide maximum stability. Mershon pointed out in 1936 that “we can move the teeth to where they should be; Nature will then move them to the place where they are best adapted to the dental balance” . To prevent this, we implement the retention process that maintains and fixes the occlusion obtained through the orthodontic correction.

As Kaplan (1) noted, up to 1860 no mention was made on the need for retention or use of retainers. Perhaps the first mention of relapse was made by Coleman in England (1865). One year later, Marvin described the physiological reasons for retention. In 1881, Smith designed what may be considered as the first retainer used in the U.S.A. which consisted of a vulcanite plate with a bar to position the upper incisors.

Since then to the present time, not much emphasis has been placed on the study of relapse, Angle in 1907 stating that retention “ is not very highly considered “ and Hahn, in 1944 calling it the “stepchild” of Orthodontics. However, the concept of how teeth should be retained has developed a lot since Angle in 1887 proposed total immobilisation so that the new bone formation would not interfere in the process. This same author, later in 1907, proposed total freedom of tooth movement with the exception of the direction towards which the tooth would naturally tend to return.

As Uhde et al. noted (2), Riedel revolutionised in 1960 all the previously accepted ideas on stability and proposed his nine retention rules. Today this concept is understood as something more dynamic and biological rather than purely mechanical: integrating into the relapse process the changes involved in craniofacial maturity that bring about similar modifications in both treated and non-treated arches. The limits of both processes are still not well defined, and in the forthcoming years research will have to clarify the implications of both in the occlusal homeostasis that develops in the dental arches as time goes by.

Nowadays, retention has to be taken into consideration in the treatment plan and, during this period, measures designed to prevent relapse have to be taken. Retention, therefore, is an active part of the therapeutic process and responsibility for it has to be assumed by both the clinic and patient, once the brace has been removed from the mouth. It is, therefore, essential to provide truthful information on the frequency of relapse, which may occur even after adequate diagnosis and treatment. By doing so, the patient will be motivated to correctly fulfilling the requirements set by the orthodontist when maintenance begins.

We still know very little about how relapse works, as Hellman said back in 1936: “we are almost completely ignorant of the factors associated with retention”.

For a long time now various schools have followed one another in the search for, and study of, the main factors of stability. Various concepts have been invoked which today have had an influence on the multifactorial approach to relapse; among them a good occlusion, as well as respect for the bimolar and bicanine widths; much importance has also been placed on the position of the lower incisor perpendicular to the basal bone, as well as to the achieving of a correct muscular balance following treatment. Lastly, the interceptive school considers that early treatment of malocclusions provides greater final stability.

Nowadays relapse is considered as a multifactorial process, as has already been stated, in which the abovementioned concepts have been accepted by traditional schools, as well as a series of conditioning factors, even though these have yet to be resear-ched in depth. As Berg and Fredlund stated in 1981 (3) “one has to recognize the difficulty of proving and quantifying the causal factors of relapse and there is a lack of scientific data to enable us to identify the origin of stability and relapse”.

## Long-term stability of lower dental alignment

Relapse in Orthodontics can be studied from the vertical, transversal and sagittal relationships of the arches, as well as in relation to rotations or arch shape; but, most studies have been based on the stability of dental alignment following orthodontic correction.

Most of what has been published on relapse of dental alignment refers to the lower arch, as traditionally this has been considered as a template that provides the form around which the maxilla develops and functions, mandibular alignment, therefore, determining the alignment of the upper arch.

The first published studies considered the bicanine diameter (and also the intermolar) as the key to the stability of anteroinferior dental alignment. It was McCauley, in 1944, who suggested that the intercanine distance had to be maintained during treatment, basing his observation on the subjective assessment of his own cases, as did Strang at around the same time and Steadman in the 1960s.

In the 1970s, King (4) claimed that expansions could be stable if they are not of great magnitude. Fastlich (5) studied 28 treated cases, and several others that were not, and associated the reduction in the bicanine diameter that appears with age with increased crowding in both groups. Likewise Johnson (6) in his work on 11 treated cases, and studied after more than six years out of the retention, concluded that there was a tendency in most of them to a reduction in the bimolar and bicanine diameters as well as an increase in crowding. This tendency towards a contraction of the intercanine width in the post-retention stage was also noted at the end of the 1970s by authors such as El Mangoury (7), Sondhi et al. (8) or, more recently, Gianelly (9) in cases treated with and without extractions, among others.

Less numerous are the studies published on long-term stability of dental crowding in cases treated with functional braces. These date back to the 1980s and arrive at similar conclusions. Hence Pancherz (10); Madone and Ingervall (11) and Hale and Tongue (12) found that during treatment the bicanine width increased, but during the retention stage it diminished at the same time as lower crowding increased. Hime and Owen (13) studied eleven cases 4.4 years after the end of active treatment with function regulators. Their results show that some of the increases in the transversal diameters achieved during treatment are maintained, but to a lesser degree than in previously published works on dental braces. Arch width diminished during the treatment and continues to diminish after the retention stage, but also to a lesser extent than that of previously published studies on dental braces. The reduced caseload presented in this study makes comparisons with other studies undertaken on treatments with dental braces very difficult, as the latter usually present larger samples.

In the 1960s, and inspired by authors such as Riedel, the University of Washington began to study orthodontic relapse, concentrating primarily on the effect on arch dimensions. Thus, Arnold studied 50 cases treated with and without extractions, following a retention period of more than 5 years, and concluded that the bicanine distance returned to its value prior to treatment regardless of the intensity of the expansion produced during that stage, as noted Blake and Bibby (14). Similar results were obtained by Welch in his study of 34 cases treated with extractions after more than 5 years out of retention, as noted by Little et al. (15).

Uhde et al. (2) reported the results of Dona. He studied 22 treated cases from 2 to 6 years out of retention and documented a marked tendency of the bicanine and bimolar widths, increased during the active retention stage, to relapse to their original values following the completion of the retention period. Likewise, he noted a reduction in the long-term arch length, as did Litowitz in his published work on the changes in position and inclination of the incisors in treated cases, as noted by Sadowsky and Sakols (16).

Peak in 1956 , noted by Little et al. (15) and Lombardi (17) and Berset (18) in the 1970s concluded that the cases in which an expansion took place during treatment relapse to values lower than those prior to the beginning of treatment. The works of Gardner et al. in 1998 are in agreement with Williams’ results in 1985 (19). They reached a similar conclusion in there study on the long-term stability of orthodontic cases. A third of Williams’ cases presented relapse of crowding as well as a reduction in the bicanine width to values lower than the preexisting ones. He considered that the essential factor in explaining the phenomena of long-term lower incisor crowding is not so much the position achieved by the teeth on completion of the active treatment stage (measured as bicanine and bimolar distances), but the remaining potential for the late growth of the maxillae which would progressively straighten out the upper incisor and subsequently the lower one, so producing crowding.

Other authors such as Walter in the 50s and 60s claim that the expansion achieved during active treatment can be maintained with the passage of time, as noted by Little et al. (15) or Ward et al. (20) . Already, in 1949, Korkhaus maintained that arch length could be improved and maintained by expansion (20).

At the end of the 1970s, Gallerano studied 83 cases treated with or without extractions after 9.6 years out of retention. His conclusions are similar to those of Walter on the maintenance of expansion achieved during active treatment, as noted by Little et al. (15).

Herberger (21) studied 56 cases treated without extractions after more than two years out of retention, concluding that the expansion of the bicanine arch achieved during treatment diminished after the retention period but with a net gain with respect to the width prior to treatment.

In 1976 Gardner and Chaconas (22) studied a sample of 103 patients after an average of 5.2 years out of retention and noted a greater reduction in arch length and bicanine diameter in cases treated with extractions compared with those in which the treat-ment was conservative.

Shapiro (23), at around the same time, reached similar conclusions on cases treated with or without extractions. The arch length diminished over time and this reduction is greater in cases of extraction. The bicanine length also diminishes in both types of treatment and the net increase or loss after some years depended on the type of malocclusion. Thus, an overall increase in intercanine arch is obtained in cases of class I and class lI-1 treated without extractions, as well as in the cases of class II-2 treated without extractions.

Then, in the 1980s, Uhde et al. (2) analyzed 72 cases treated with or without extractions between the ages of 12 and 35 years old, noting the tendency of the teeth to return to positions prior to treatment, but also to maintain most of the correction during that treatment.

Sadowsky and Sakols (16) reached similar conclusions in their study of 96 patients treated with or without extractions after a mean of 20 year post-retention. They too considered that there was a tendency in both arches to crowding after treatment, slightly greater in the lower arch, but did not find differences between the different malocclusion groups either in cases treated with or without extractions.

For more than 35 years, Dr. Little’s group (24,25), at the University of Washington has been compiling diagnostic records of more than 600 cases treated there proposing a strongly documented approach to the problem of lower incisor crowding both in cases treated and in normal occlusions without treatment (26,27). They have published studies on cases treated with extractions of premolars in permanent dentition after ten and twenty years out of retention, as well as with serial extraction of first and second premolars. Differences have been assessed between cases treated with early and late extractions of first premolars. Relapse of lower crowding has also been studied in cases treated with extractions both when the arch length was sufficient and when it was inadequate. They have also worked on the long-term development of very proclined incisors following combined orthodontic and surgical treatment in adults with Class lII, as well as on relapse following treatment with extractions of lower incisors.

The conclusions arrived at from all these studies both in cases treated and in non-treated normal occlusions, are as follows:

-Arch length progressively decreases.

-Arch width , measured as bicanine diameter, also gradually decreases. In the case of patients treated, this tendency is apparent whether this dimension has been increased, maintained or reduced during the active treatment stage.

-There is a tendency towards lower incisor crowding with the passage of time that continues up to the age of 40 and probably after.

-The degree of lower incisor crowding is unpredictable and variable. No variables extracted from clinical, biometric or cephalo-metric findings have been found that could provide good predictors of the future behaviour of anterior alignment.

Furthermore, very few differences have been recorded in the long-term result of the treatment between cases treated with extractions of first or second premolars, whether these were undertaken on permanent dentition or mixed dentition (serial extraction).

Glenn et al. (28) studied the long-term stability of 28 cases treated without extractions, reaching conclusions similar to those presented by the Washington group. They did not consider, moreover, the analyses of Peck and Bolton to be suitable for relating the vestibulolingual and mesiodistal diameters of the incisors to the irregularity index, either before or after treatment. Freitas et al. (29) arrived at similar conclusions in their study of the Peck index in relation to the post-retention stability on 56 cases treated with four premolar extractions and evaluated 5 years out of retention.

The conclusion reached by this research group (24-28) is that the changes that occur over time both in normal untreated occlusions and in orthodontic cases are of a similar nature, but to a different degree. Hence, the tendency to a reduction in arch size and lower incisor crowding is maximum in cases treated with extractions and minimum in normal occlusions. This different behaviour may have its origin in the magnitude of the crowding at the beginning of treatment, which will also be maximum in cases of extractions, intermediate in conservatively treated cases and minimum in untreated individuals.

Sinclair and Little (26,27) put forward the hypothesis that orthodontic treatment acts as an accelerator of the future changes that, physiologically, the process of craniofacial maturing would produce in the arches. It is, therefore, logical that in present studies on relapse that the treated cases present lower stability than untreated ones, although, perhaps, if those same individuals are studied in forthcoming years, the results may be very similar in the two groups.

This hypothesis clearly shows the importance systematically keeping records on patients outside retention. It would be of great interest to evaluate studies on the changes undergone in treated dental arches after more than twenty years out of retention; only in this way will we be able to clearly understand the pathogenesis of relapse in Orthodontics.

## Long-term stability of upper tooth alignment 

As has already been commented upon, studies on the stability of upper dental alignment following orthodontic treatment are much less numerous than those referring to the lower arch.

Many of them refer to the relapse of dental rotations and others include the study of upper crowding in works on stability in both arches. Hence, Sadowsky and Sakols (16) and Uhde et al. (2) also observe a tendency towards crowding in the upper arch following the retention period, both in cases treated with extractions and in those without them. They found a modest but significant correlation between the increase in bicanine diameter during treatment and crowding following the retention period, even though an overall increase compared to the initial situation takes place. In none of their cases did a crowding greater than the ideal range, arbitrarily established by them of between 0 and 3 mm, appear.

In 1986 Allred, at the University of Washington and employing this same method, studied the long-term stability of upper anterior alignment in patients treated with extractions of premolars more than 9.5 years after the retention period was over, aided by cephalometric models and measurements. The sample consisted of 31 cases with different malocclusions. The results showed that upper incisors tended towards crowding following retention, but showed greater stability than the lower ones. Likewise, an overall improvement in upper alignment took place compared with before beginning treatment. Upper incisor alignment before treatment is not a reliable predictor of what is going to happen in the post-retention stage. Neither did they find significant correlations between stability of the upper incisor alignment following the retention stage and clinical variables such as age, gender, Angle class or retention time; nor biometric ones such as arch length and width, overjet, overbite or irregularity index, either before or after treatment. However, they did find a significant correlation of a cephalometric kind: the upper incisor crowding is greater in cases in which a straightening of the same has taken place during treatment, as noted by Canut and Arias (30)

Fayos et al, in a 2004 study on 30 cases treated with or without extractions after post-retention periods of between 3 and 14 years, concluded that relapse is greater and more frequent in the lower arch as opposed to the upper one and did not find any factor significantly related with late irregularity; as noted by Paredes et al. (31)

In 2010 Park et al. (32) worked on a sample of 96 patients (51 adolescents and 45 adults) assessed following a period of 16 years out of retention. They concluded that relapse of all biometric parameters is the rule, lower irregularity being greater than upper irregularity and the changes of a more marked character in the adolescent subgroup.

At around the same time, Quaglio et al. (33), on a sample of 70 patients treated with extractions, concluded that upper incisor stability is high and that there were no important differences between initial malocclusions or types of extraction (two as opposed to four premolars).

In 2011, López-Areal et al. (34) evaluated the long-term stability of upper arch alignment in orthodontically treated patients. They analyzed pre-treatment, post-treatment and post-retention models (average 5 years) of 51 patients treated with or without extractions. They conclude that upper incisor crowding relapses, although they noted a net improvement with respect to the initial stage, both in cases treated with or without extractions. The arch length also relapses in both cases. Intercanine and intermolar widths as well as overjet and overbite are stable in the long term. They stated that the long-term response of maxillary incisor alignment is unpredictable, as they did not find any relationship with initial alignment, gender, age, type of malocclusion or type of treatment employed. Only the irregularity index at the end of treatment presented a significant correlation with late irregularity, so indicating that an excellent termination of cases is the determining factor in the long-term stability of upper incisors.

## Final Considerations

Having reviewed the published literature, it is clear that post-retention changes in the upper arch are similar to those found in the lower arch, i.e. a progressive tendency towards crowding and to the reduction in arch sizes, as the studies on the long-term development of untreated arches show: Sinclair and Little (27), Bishara et al. (35,36), Helm and Petersen (37).

Most authors consider that post-retention crowding is variable and unpredictable as they do not find any clinical, biometric or cephalometric variables that may predict their future development. Several authors refer to slightly significant correlations with parameters such as bicanine distance Uhde et al. (2), the straightening of upper incisors (Allred); initial crowding (Nanda in 1995 and more recently, in 2005, Ormiston et al, as noted by Park et al. (32) or crowding following treatment López-Areal et al. (34).

The physiological tendency of the arches is towards progressive reduction of their dimensions and an increase in incisor crowding. These changes are temporarily detained with orthodontic treatment but when the treatment is finished, the maturing process of the arches is reinitiated with greater virulence than in cases that were never treated.

Most post-retention changes occur during the first ten years and require long-term studies in order to be able to assess relapse as a whole in treated arches treated and maturity changes in untreated cases.

The only way of ensuring good incisor alignment in the long term is permanent or semi-permanent retention. Most of the studies undertaken, like those published by Årtun, Riedel, Little, Nett and Huang, Destang and Kerr, advocate retention for long periods of time or when less up until growth has finished like Zachrisson’s, although there are other authors who advocate not using them in most cases like Aasen and Espeland in 2005 (38). Studies like those published by Pandis et al. in 2007 (39) and Booth et al. in 2008 carried out on the periodontal effects of permanent braces are reassuring (40), but regular controls of plaque and periodontal treatment may be necessary in susceptible patients.

It is furthermore necessary to assess whether retention over long periods of time ensures long-term stability or only postpones relapse.

Similarly, studies of this type on patients treated with functional braces would be interesting, as, to date, there have been very few of these.

## References

[1] Kaplan H (1988 ). The logic of modern retention procedures. Am J Orthod Dentofacial Orthop.

[2] Uhde MD, Sadowsky C, BeGole E (1983). Long-term stability of dental relationships after orthodontic treatment. Angle Orthod.

[3] Berg R, Fredlund A (1981). Evaluation of orthodontic treatment results. Eur J Orthod.

[4] King EW (1974). Relapse of orthodontic treatment. Angle Orthod.

[5] Fastlicht J (1970 ). Crowding of mandibular incisors. Am J Orthod.

[6] Johnson KC (1977). Cases six years postretention. Angle Orthod.

[7] El-Mangoury NH (1979 ). Orthodontic relapse in subjects with varying degrees of anteroposterior and vertical dysplasia. Am J Orthod.

[8] Sondhi A, Cleall JF, BeGole EA (1980 ). Dimensional changes in the dental arches of orthodontically treated cases. Am J Orthod.

[9] Gianelly AA (2003). Arch width after extraction and nonextraction treatment. Am J Orthod Dentofacial Orthop.

[10] Pancherz H (1977 ). Relapse after activator treatment. A biometric, cephalometric, and electromyographic study of subjects with and without relapse of overjet. Am J Orthod.

[11] Madone G, Ingervall B (1984). Stability of results and function of the masticatory system in patients treated with the Herren type of activator. Eur J Orthod.

[12] Hale LRO, Tongue EA (1986). A long-term follow-up of four cases treated with the Andresen appliance. Br J Orthod.

[13] Hime DL, Owen AH (1990 ). The stability of the arch-expansion effects of Fränkel appliance therapy. Am J Orthod Dentofacial Orthop.

[14] Blake M, Bibby K (1998 ). Retention and stability: a review of the literature. Am J Orthod Dentofacial Orthop.

[15] Little RM, Wallen TR, Riedel RA (1981 ). Stability and relapse of mandibular anterior alignment-first premolar extraction cases treated by traditional edgewise orthodontics. Am J Orthod.

[16] Sadowsky C, Sakols EI (1982 ). Long-term assessment of orthodontic relapse. Am J Orthod.

[17] Lombardi AR (1972 ). Mandibular incisor crowding in completed cases. Am J Orthod.

[18] Berset A (1972). A stability of the lower dental arch after orthodontic treatment. Trans Eur Orthod Soc.

[19] Gardner RA, Harris EF, Vaden JL (1998 ). Postorthodontic dental changes: a longitudinal study. Am J Orthod Dentofacial Orthop.

[20] Ward DE, Workman J, Brown R, Richmond S (2006). Changes in arch width. A 20-year longitudinal study of orthodontic treatment. Angle Orthod.

[21] Herberger RJ (1981). Stability of mandibular intercuspid width after long periods of retention. Angle Orthod.

[22] Gardner SD, Chaconas SJ (1976). Posttreatment and postretention changes follwoing orthodontic therapy. Angle Orthod.

[23] Shapiro PA (1974). Mandibular dental arch form and dimension. Treatment and postretention changes. Am J Orthod.

[24] Little RM (1990). Stability and relapse of dental arch alignment. Br J Orthod.

[25] Little RM (1999 ). Stability and relapse of mandibular anterior alignment: University of Washington studies. Semin Orthod.

[26] Sinclair PM, Little RM (1983 ). Maturation of untreated normal occlusions. Am J Orthod.

[27] Sinclair PM, Little RM (1985 ). Dentofacial maturation of untreated normals. Am J Orthod.

[28] Glenn G, Sinclair PM, Alexander RG (1987 ). Nonextraction orthodontic therapy: posttreatment dental and skeletal stability. Am J Orthod Dentofacial Orthop.

[29] de Freitas KM, Janson G, de Freitas MR, Pinzan A, Henriques JF, Pinzan-Vercelino CR (2007). Influence of the quality of the finished occlusion on postretention occlusal relapse. Am J Orthod Dentofacial Orthop.

[30] Canut JA, Arias S (1999 ). A long-term evaluation of treated Class II division 2 malocclusions: a retrospective study model analysis. Eur J Orthod.

[31] Paredes V, Gandia JL, Cibrian R (2006). Determination of Bolton toot-size ratios by digitalization, and comparison with the traditional method. Eur J Orthod.

[32] Park H, Boley JC, Alexander RA, Buschang PH (2010). Age related long-term posttreatment occlusal arch changes. Angle Orthod.

[33] Quaglio CL, de Freitas KMS, de Freitas MR, Janson G, Henriques JFC (2011). Stability and relapse of maxillary anterior crowding treatment in Class I and Class II Division 1 malocclusions. Am J Orthod Dentofacial Orthop.

[34] López-Areal L, Paredes V, Gandía JL (2011 ). Long-term analysis of upper incisor crowding. A longitudinal study orthodontically treated patients. Med Oral Patol Oral Cir Bucal.

[35] Bishara SE, Jakobsen JR, Treder J, Novak A (1998). Arch length changes from 6 weeks to 45 years. Angle Orthod.

[36] Bishara SE, Jakobsen JR, Treder J, Nowak A (1997 ). Arch width changes from 6 weeks to 45 years of age. Am J Orthod Dentofacial Orthop.

[37] Helm S, Petersen PE (1989 ). Individual changes in malocclusion from adolescence to 35 years of age. Acta Odontol Scand.

[38] Aasen TO, Espeland L (2005). An approach to maintain orthodontic alignment of lower incisors without the use of retainers. Eur J Orthod.

[39] Pandis N, Vlahopoulos K, Madianos P, Eliades T (2007). Long-term periodontal status of patients with mandibular lingual fixed retention. Eur J Orthod.

[40] Booth FA, Edelman JM, Profitt WR (2008). Twenty-year follow-up of patients with permanently bonded mandibular canine-to-canine retainers. Am J Orthod Dentofacial Orthop.

